# Cultural adaptation of the mental health first aid guidelines for assisting a person at risk of suicide to China: a Delphi expert consensus study

**DOI:** 10.1186/s12888-020-02858-9

**Published:** 2020-09-16

**Authors:** Shurong Lu, Wenjing Li, Brian Oldenburg, Yan Wang, Anthony F. Jorm, Yanling He, Nicola J. Reavley

**Affiliations:** 1 Jiangsu Provincial Centre for Disease Control and Prevention, Nanjing, 210009 China; 2 Nossal Institute for Global Health, Melbourne School of Population and Global Health, University of Melbourne, Melbourne, Victoria 3000 Australia; 3 Centre for Mental Health, Melbourne School of Population and Global Health, University of Melbourne, Melbourne, Victoria 3010 Australia; 4 Shanghai Mental Health Centre, Shanghai, 200030 China

**Keywords:** Suicide, Mental health first aid, Cultural adaptation, Delphi study, China

## Abstract

**Background:**

Suicide is a significant public health concern in China and there is a need for evidence-based suicide prevention programs to assist people in the community who may be in a position to support those in their social networks who are at risk of suicide. English-language mental health first aid guidelines for this purpose have been developed. However, due to differences in culture, language and health systems, guidelines for English-speaking countries require cultural adaptation for use in China.

**Methods:**

A Delphi expert consensus study was conducted among mainland Chinese panellists with a diverse range of expertise in suicide crisis intervention (*n* = 56). Using the mental health first aid guidelines used in English-speaking countries as a basis, a questionnaire containing 141 statements on how to help a person at risk of suicide was developed and translated. Panellists were asked to rate the importance of each item for inclusion in the Chinese guidelines. They were also encouraged to suggest any additional statements that were not included in the original questionnaire. Statements were accepted for inclusion in the adapted guidelines if they were endorsed by at least 80% of panellists as essential or important.

**Results:**

Consensus was achieved after two survey rounds on 152 statements for inclusion in the adapted guidelines for China, with 141 adopted from the guidelines for English-speaking countries and 11 generated from the comments of panellists.

**Conclusions:**

While the adapted guidelines were similar to the guidelines for English-speaking countries, they also incorporated actions specific to the Chinese context, including Chinese attitudes towards suicide, the role of families and friends and removal of the means of suicide. Further research is needed to investigate the use of the guidelines by the Chinese public and the implementation of Mental Health First Aid training in appropriate settings in China.

## Background

Suicide represents a major societal and public health problem [1]. It is estimated that over 800,000 people die by suicide every year, with 75.5% of these deaths occurring in low- and middle-income countries (LMICs) [2]. Furthermore, for each suicide, there are more than 20 suicide attempts [2, 3]. Each suicide is not only an individual tragedy, but also has a profound impact on society by indirectly affecting many individuals, including families, friends, colleagues and people in the wider community [4].

In contrast to Western countries, there is no strong religious or legal prohibition against suicide in China; instead, some traditional Chinese cultural values incorporate a view of suicide as being morally justifiable. For example, Confucianism advocates giving up life in certain circumstances to achieve the dignity and purity of the soul (*she sheng qu yi*, in Chinese); while in Buddhism, death marks the transition from this life to the next for the deceased [5] . Some evidence suggests that Chinese people who follow Buddhism are more likely to view suicide as acceptable [6]. In the 1990s, China had one of the highest rates of suicide in the world (23 per 100,000 and a total of 287,000 suicide deaths per year), with an unusual epidemiological pattern (viz., the suicide rate in women was 25% higher than that in men, and rural rates were three times higher than urban rates) [7]. However, subsequent decades have seen a significant decline in the suicide rates in China [8–10]. It is likely that this is related to socioeconomic changes, such as economic development, rapid urbanization, improved emergency medical systems, improved transportation conditions and reduced availability of lethal pesticides in rural areas [10, 11].

Nevertheless, data show that the positive impact of these factors on suicide rates is gradually diminishing, as the pace of decline has slowed in recent years [8–10]. Epidemiological studies have shown that, despite a reduction in overall suicide rates, the rates in young males and older adults have remained high and may have even increased among the elderly, particularly in rural areas. Nearly 80% of completed suicides in China occurred among rural residents, with more than 40% among those aged 65 or above [9]. Recent rapid changes in Chinese society may also have contributed to rising suicide rates, particularly among those with insufficient social support and healthcare [12]. These include the aging of the population [13], large-scale migration of workers from rural to urban areas [11], “left-behind children” in rural areas [13], and the global economic recession [14]. Thus, suicide remains a significant social and public health concern in China, and evidence-based suicide prevention programs are desperately needed.

However, as in many other LMICs, suicide prevention efforts are relatively limited in China, mostly depending on health professionals for recognition and clinical management. Some strategies targeting practice changes in mental health services (e.g., improved ward safety, community services, and staff training) have had beneficial impacts on suicide in high-income countries [15, 16], but evidence suggests that most Chinese people at risk of suicide have limited access to appropriate health services [17, 18]. Community education programs, including gatekeeper training, which involves educating community members to recognise and identify people at risk of suicide and to assist them in receiving appropriate care are also commonly implemented in high-income countries [19].

One such community-based education program is Mental Health First Aid (MHFA) training. This training program teaches members of the public how to provide mental health first aid, which has been defined as “the help offered to a person developing a mental health problem, experiencing a worsening of an existing mental health problem or in a mental health crisis; the first aid is given until appropriate professional help is received or until the crisis resolves” [20]. MHFA training has been shown to be effective in improving mental health first aid knowledge, the ability to recognise a mental disorder, confidence and intentions to provide mental health first aid, and the amount of help provided [21]. Several studies conducted in Chinese-speaking communities in Hong Kong and Australia have found similar effects [22, 23]. The content of the English-language version of MHFA training has been informed by Delphi expert consensus studies used to develop guidelines for someone providing mental health first aid, including to a person at risk of suicide [24], developing depression [25] or psychosis [26].

Given the differences between China and English-speaking countries in culture, language, health systems and available resources for suicide prevention, the suitability of mental health first aid guidelines developed for English-speaking countries for use in China is currently unknown [20] and cultural adaptation is warranted [27]. Therefore, we conducted this Delphi expert consensus study to adapt the mental health first aid guidelines for assisting a person at risk of suicide used in English-speaking countries for China, a country with a very different health system and cultural understanding of suicide [28, 29]. The Delphi method of assessing expert consensus offers a systematic way to incorporate practice-based evidence with evidence-based practice in order to enable recommendations and decisions to be made [30, 31]. Ultimately, this adaptation study is expected to provide a set of culturally, contextually and linguistically appropriate statements describing information members of the public and frontline workers should have and actions they can take to help a person at risk of suicide in China.

## Methods

A two-round Delphi expert consensus survey was conducted among an expert panel comprising mental health professionals, Hotline operators and consumers and carers from China. The panel of Chinese experts was invited to rate the importance of statements in the guidelines used in English-speaking countries for inclusion in the guidelines for China. Statements that achieved substantial consensus by the panel were considered to be recommended actions for members of the public to assist a person at risk of suicide in China. The panellists were encouraged to suggest any additional actions that were not included in the original questionnaire and, in particular, to suggest statements that were culturally specific. These suggestions were used to generate new statements.

**Fig. 1:**
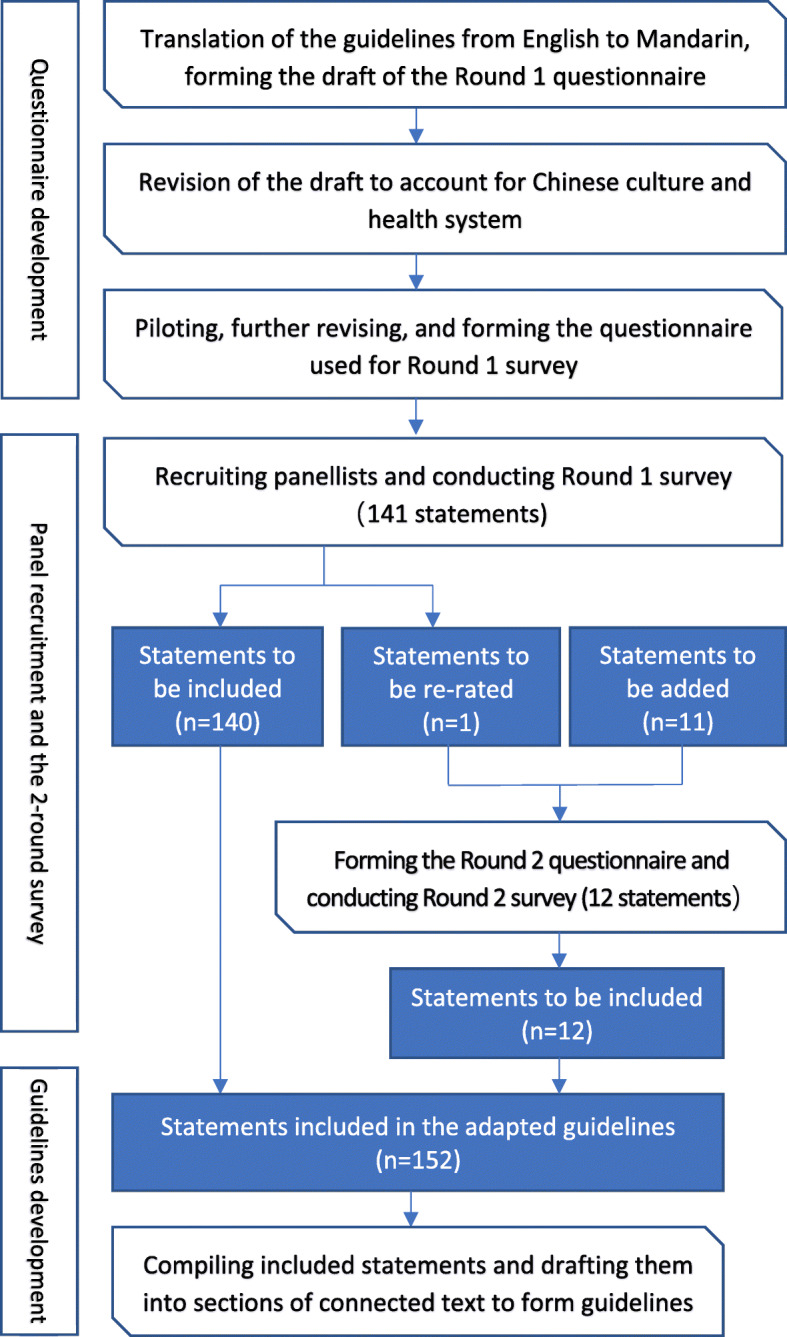
Overview of the Delphi process

This Delphi study involved four stages: (1) Questionnaire development for the Round 1 survey; (2) Panel identification and recruitment; (3) Data collection over 2 survey rounds; and (4) Guidelines development. The steps taken in each stage and the number of statements involved in each round of the survey are shown in Fig. 1.

### Questionnaire development for the round 1 survey

The questionnaire for Round 1 was developed from the translation of the mental health first aid guidelines for assisting a suicidal person used in English-speaking countries, which were revised and updated in 2014 [24]. The translation was conducted by a professional translator and edited by authors who speak both English and Chinese (SL, WL, YH and WY) and have an understanding of the mental health system in China to be more appropriate to this context. As an example of this, expressions in the English guidelines of “*a hospital emergency department*”, “*an emergency number*” or “*to see a doctor*” were replaced by “*an emergency department of a specialised mental health institute*” or “*hotline of psychological crisis intervention*” in the Chinese questionnaire, where applicable, as these are the available services for suicide in China. Wording changes were also made to improve readability and clarity or to reduce ambiguity.

Then, a copy of this questionnaire was presented to 16 people, who were native Chinese speakers but completely independent of the study. They were asked to comment on the clarity, non-ambiguity and readability of each statement included in the questionnaire and to give suggestions on how to improve them. The questionnaire was further revised based on these comments and suggestions.

Finally, 141 original helping statements were included in 7 sections of the Round 1 questionnaire (see Table 1 in the Additional file). Statements in this questionnaire were presented to panellists, who were invited to rate their importance for inclusion in the mental health first aid guidelines for members of the public in China to assist someone at risk of suicide.

### Panel identification and recruitment

In this study, in addition to mental health professionals and consumers and carers, we also identified Hotline operators in China as potential eligible experts for this study due to their frequent involvement in providing telephone support to people in a suicidal crisis and their familiarity with mental health services for suicide in China [32, 33].

Hotlines are currently operated in multiple urban regions in China (mostly metropolitan cities such as Beijing, Shanghai and Guangzhou). Hotlines for telephone crisis support (variously covering suicidal thoughts or attempts; trauma; stresses from work, family or society) have become more common in China in the last decade, and are relatively well known for their anonymity, reliability and quality of support provided [33, 34]. The Chinese government plans to set up at least one Hotline in each provincial region across the country by 2020 [35].

In this study, we recruited operators from the Hotlines of Shanghai (*Shanghai Hotline for Psychological Aid,* http://www.12320-5.org.cn/) and Beijing (*Beijing Psychological Aids Hotline,* http://www.crisis.org.cn/Home), which are two of the earliest established and most recognised Hotlines in China. Operators working for the two Hotlines need to undertake extensive training and meet further selection criteria (e.g. hold a recognized psychological counseling certificate), to ensure that they are confident, skilled and able to provide support to Hotline’s callers. Some of these operators also engage in activities that give them a broader exposure to people’s suicidal experiences [33], such as offline group activities among families and friends of people who have died by suicide.

To be eligible for participation, experts needed to meet the following criteria: be aged over 18 **AND** with at least 9 years of education, **AND**

1. For mental health professionals
  To have been working in a specialised mental health institute for at least 2 years, **AND**
  To have been involved in suicide crisis management.
2. For hotline operators
  To have been working as an operator for at least 1 year for the Hotline operated in Shanghai or Beijing, **AND**
  To have experience in telephone suicide crisis intervention.
3. For consumers and carers

- To have relevant lived experience of suicidality or taking care of a suicidal person on a daily basis, **AND**
- To have had enough knowledge on (self-)management of suicide, which was judged by the researcher (SL) through verbal communication and clinical observation, **AND**
- To have an adequate ability to read and write, as well as an adequate understanding of how to complete the survey online.

An initial recruitment advertisement was sent out via email to mental health professionals and Hotline operators. A snowball recruitment method was used among these two groups of experts and they were encouraged to send the email on to other eligible professionals or operators they knew. Potential consumers and carers were recruited from the Clinical Psychological department in the Suzhou Guangji Hospital, a specialised mental health institute in Jiangsu Province of China.

As suicide is such a sensitive topic in Chinese society and few suicide advocacy organisations or support networks exist, we were not able to recruit a minimum of 23 consumers/carers as recommended for a separate expert panel [54], therefore we combined professionals, Hotline operators, consumers and carers into one panel.

### Data collection and analysis

Recruited panellists were sent a link and a quick response code, both of which led them to an online questionnaire hosted by Questionnaire Star (*Wen Juan Xing*, https://www.wjx.cn/) which they could access via a computer or mobile phone (by WeChat – a commonly used mobile application for social interaction in China). Participants received a clear explanation of the purpose of the study. They were asked to choose their primary/major occupation and to rate each statement on a 5-point Likert scale (1 = Essential, 2 = Important, 3 = Don’t know/It depends, 4 = Unimportant, 5 = Least important) according to how important they believed it was for inclusion in the guidelines for a member of the public providing mental health first aid to a suicidal person in China. In Round 1, panellists were also encouraged to provide comments on existing statements or to suggest new helping actions that were not covered in the questionnaire.

In line with previous similar studies in English-speaking countries [24, 25, 36, 37], statements were immediately included in the guidelines if they were endorsed by ≥80% of panellists as either essential or important. Statements were re-rated in the following round if they were rated as essential or important by 70–79% of the panel. Statements were immediately excluded from the guidelines if they were rated as essential or important by less than 70% of the panel.

Comments collected were categorised and translated into English and then reviewed by the working group (SL, WL, YH, NR, WY). Suggestions that contained novel ideas were used to create new statements to be included in the questionnaire of the subsequent Round 2 survey. Statements from Round 1 that met the criteria to be re-rated (i.e. being rated as essential or important by 70–79% of the panel) were also included in the Round 2 questionnaire.

Following the first-round, panellists were sent a report containing a summary of the overall ratings for the statements, as well as their ratings for each statement. This allowed the panellists to compare their ratings with the level of endorsement given by the group as a whole in order to inform their future ratings for those statements that needed to be re-rated.

### Guidelines development

Endorsed statements (i.e. those being rated as either essential or important by ≥80% of the panel) from the two survey rounds were compiled and drafted into sections of connected text. Where possible, statements were combined and repetition deleted. Statements that received comments suggesting ambiguity in the interpretation of their meaning were re-worded to make them clear and easy to understand. The draft was then circulated to members of the working group (SL, WL, YH and WY) who were native Mandarin-speakers to finalise structure and wording, creating a set of guidelines that were written in plain Mandarin and could easily be followed by members of the public in China. A number of iterations were circulated and completed before the group agreed on the final guidelines. See the full document of the guidelines in the Additional file 2.

## Results

### Expert panel information

A total of 56 expert panellists (women 75%) completed the Round 1 survey, and 41 of them participated in Round 2 (retention rate = 73%). The sociodemographic characteristics of all panellists are shown in Table 1. The participants were aged 28–67 years (mean ± sd = 41.5 ± 9.4, median = 39), and all of them, except for two consumers/carers, had at least a university level of education. Panellists were from three provincial regions of China, i.e. Shanghai (*n* = 30, 54%), Beijing and Jiangsu (*n* = 13, 23%, respectively).

**Table 1:** Characteristics of participants (*n* = 56)

Item	Category	N	%
Gender	Men	14	25.0
	Women	42	75.0
Education	Primary/Secondary	2	3.6
	University or above	54	96.4
Region	Shanghai	30	53.6
	Beijing	13	23.2
	Jiangsu	13	23.2
Occupation	Mental health professionals	23	41.1
	Psychiatrists	10	17.9
	Psychotherapists	5	8.9
	Psychiatric nurses	3	5.4
	Psychological counsellors	3	5.4
	Other mental health workers^a^	2	3.6
	Hotline operators	28	50.0
	Consumers/Carers	5	8.9
Age (years)	Range	28–67	
	Mean ± sd	41.5 ± 9.4	
	Median	39.5	
Years of experience in suicide intervention	Range	1–33	
	Mean ± sd	8.7 ± 8.1	
	Median	5	

^a^Including one social worker and one community mental health worker

Of these panellists, 23 were mental health professionals, including psychiatrists (*n* = 10), psychotherapists (*n* = 5), psychiatric nurses (*n* = 3), psychological counsellors (*n* = 3) and 2 other professionals (1 social worker and 1 community mental health worker). Another 28 were Hotline operators and 5 were consumers with lived experience of suicidality or their carers. These experts had 8.7-year experience in suicide intervention on average (range = 1–33, median = 5). Some of the panellists had multiple roles in the field of suicide intervention. For example, most mental health professionals in this study also conduct research and some Hotline operators were also qualified psychological counsellors. See Table 1 for details.

### Ratings of statements

Consensus was achieved on 152 statements for inclusion in the adapted suicide mental health first aid guidelines for China after two survey rounds (see Table 2 of the Additional file for a full list of these included statements). These endorsed statements covered seven main topics in providing mental health first aid to a suicidal person in China, i.e.: (1) Identification of suicide risk and approaching the person; (2) Assessing the seriousness of the suicide risk; (3) Initial assistance; (4) Talking with a suicidal person; (5) Safety planning; (6) What the first aider should know; and (7) Confidentiality.

The numbers of statements to be endorsed, re-rated and added throughout the two rounds are given in Fig. 1. In all, 141 statements were rated in the Round 1 survey, with 140 being endorsed and 1 being re-rated (‘*If the first aider thinks someone might be having suicidal thoughts and feels unable to ask them, the first aider should find someone who is able to ask’,* endorsement rate = 71%) (see Table 1 of the Additional file for ratings of these statements). This statement, together with another 11 new statements developed from panellists’ comments collected through the first-round survey, was further rated in Round 2 and all of them were endorsed (see Table 3 of the Additional file for a list of these statements and their ratings).

### Differences between the guidelines for China and English-speaking countries

As well as including all the 141 statements of the guidelines for English-speaking countries, the adapted guidelines for China further incorporated 11 new statements, which were developed from text comments of panellists and were endorsed. Specifically, the 11 new statements in the adapted guidelines were:

- For the section ‘Assessing the seriousness of the suicide risk’ (*n* = 3)
  1. *The first aider should ask the person about their attitudes towards suicide.*
  2. *The first aider should ask the person if they have discussed their suicidal ideation with anyone else.*
  3. *The first aider should ask the person what they want to achieve by attempting suicide.*
- For the section ‘Initial assistance’ (*n* = 3)
  4) *The first aider should ask the person about their living situation (*e.g. *if they live alone, or with the spouse, parents or friends).*
  5) *The first aider should ask the person about other social support, such as trusted relatives or friends.*
  6) *If the suicidal person has the means to carry out their suicide plan, the first aider should take these away without the person’s permission.*
- For the section ‘Talking with a suicidal person’ (*n* = 1)
  7) *The first aider should let the person have some time alone in a safe place.*
- For the section ‘Safety planning’ (*n* = 2)
  8) *The first aider should write down the safety plan and leave it with the suicidal person.*
  9) *The first aider should ask the person to make a verbal or written agreement with the safety plan.*
- For the section ‘Confidentiality’ (*n* = 2)
  10) *The first aider should limit the discussion of suicide risk of the person to the relevant people (*e.g. *the legal guardian of the person, mental health professionals, or the police if applicable).*
  11) *The first aider should not agree to keep the person’s criminal conduct confidential.*

## Discussion

Through a two-round Delphi expert consensus survey, we culturally and linguistically adapted for China the mental health first aid guidelines for assisting a person at risk of suicide used in English-speaking countries. All initial statements in the guidelines for English-speaking countries were adopted in the adapted guidelines for China, suggesting a wide agreement on providing mental health first aid to a person at risk of suicide between China and English-speaking countries. However, as with the culturally adapted guidelines for providing mental health first aid to a person with depression in China [38], the adapted guidelines for helping a person at risk of suicide, also incorporated some new actions specific to the Chinese context.

### Attitudes towards suicide

The new statement “*The first aider should ask the person about their attitudes towards suicide*” was endorsed as being important in assessing the seriousness of suicide risk in China. Evidence suggests that attitudes towards suicide may be causally linked with suicidal behaviour, with positive attitudes accelerating the process from suicidal ideation to the eventual suicide (suicidal plan, preparation, attempt and suicide), or with negative attitudes decreasing the risk of suicide by prompting individuals to seek help [39]. A recent scoping review on mental health literacy in China found that a relatively large number of studies explored the attitudes towards suicide in China in recent decades [40], and findings of these studies suggest that, as in Western countries, attitudes towards suicide among both the general population and people with mental illness are closely associated with suicide (attempts or ideation) among Chinese people [18, 41]. Therefore, it may be important to ask about the person’s attitudes towards suicide when providing mental health first aid to a Chinese person at risk of suicide.

As already noted above, some Confucian and Buddhist traditions may lead to a view of suicide as acceptable in some circumstances and evidence shows that it is common for Chinese people to be neutral, tolerant or sympathetic to suicide [29]. In some cases, such as drug dependence, incurable illness (physically or mentally), relationship conflicts (commonly domestic), and limited family or other support during later life (prominent among rural elderly), suicide can be considered acceptable behaviour in Chinese society [18, 29]. However, in other circumstances, there remains considerable stigma towards suicide in China. For example, beliefs such as “suicide is a selfish or irresponsible act”, “suicide results in disgrace (*Diu Lian*, in Mandarin) for the family” are common [18, 29]. So, it is not surprising for Chinese panellists to suggest the new statement “*The first aider should limit the discussion of suicide risk of the person to the relevant people*”, which may reflect the emphasis on confidentiality in Chinese society. Thus, it appears to be necessary for the first aider to consider issues of potential confidentiality and stigma when providing assistance to a person at risk of suicide in China.

However, it is important to bear in mind that an individual’s attitudes towards suicide can be multifaceted and complex, beyond that defined simply as “positive” or “negative”. In the two currently most popular scales used for measuring attitudes to suicide in China – *Suicide Attitudes Questionnaire* [42], and *the Scale of Public Attitudes about Suicide* [43] - there are 29 and 47 items involved, respectively, reflecting a spectrum of attitudes. Therefore, future efforts are needed to explore whether it is possible for a first aider to explore a person’s attitudes towards suicide in this context.

Studies in multiple cultures have shown that many people, including healthcare professionals, often believe that people who are suicidal are attention-seeking, weak, cowardly, crazy, or manipulative [44, 45]. In this study, Chinese experts suggested the new statement “*The first aider should ask the person what they want to achieve by attempting suicide*”. The reasons for the endorsement of this statement were not assessed, but it may reflect a belief that some people may use suicide to influence people or circumstances around them.

### The role of families and friends

The very important role of those in a person’s social network in providing mental health first aid, particularly families and close friends, was reflected by the inclusion of two statements related to providing initial assistance, i.e. “*The first aider should ask the person about their living situation (e.g. if they live alone, or with the spouse, parents or friends)*” and “*The first aider should ask the person about other social support, such as trusted relatives and friends*”. Since the *Reform and Opening Up Policy* (i.e. the policy of “domestic reform and opening up to the outside world” that China began to implement since the year of 1978) in China, broader social networks (e.g. work, social activity, entertainment) have grown due to increased mobility [32]; still, the traditional social network consisting mainly of family members and relatives (and sometimes close friends) is considered the most important social support resource in Chinese society, particularly in times of hardship or crisis. Therefore, it is recommended that the first aider should ask about the person’s existing social network. This is somewhat different from the guidelines used in English-speaking countries, as in similar circumstances, they are more likely to advise the involvement of public services (e.g. doctors, mental health professionals, crisis teams) [24], which are less widely available in China [46].

### Removal of access to the means of suicide

Panellists proposed the new statement “*If the suicidal person has the means to carry out their suicide plan, the first aider should take these away without the person’s permission*”, and it was subsequently highly endorsed (endorsement rate = 95.1%). This might be explained by the common belief among Chinese that “A human life is of greater value than anything (*ren ming da yu tian*, in Mandarin)”, which may mean that, in the case of a person at risk of suicide, it is not necessary to show respect to the person by asking for permission [29, 47, 48]. Another possible reason could be the common occurrence of impulsive suicide attempts (e.g. taking pesticide or jumping, without proximal planning or preparations) in Chinese society, leading to a high endorsement of removing access to the means of suicide as the most effective way to solve the crisis [17]. However, the possible consequences of this action and feasibility of doing this, as well as the physical security of the first aider, need further consideration.

Meanwhile, another two related statements from the guidelines used in English-speaking countries “*If the suicidal person has the means to carry out their suicide plan, the first aider should seek the permission of the suicidal person to take them away*” and “*If the suicidal person does not agree to give the first aider the things they intend using to kill themselves, the first aider should call a mental health centre or Hotline for crisis intervention and ask for advice on the situation*” were also endorsed (endorsement rate was 96.4 and 85.7%, respectively). To avoid any confusion, it is equally important for these interrelated actions to be considered and interpreted as a whole in any training course developed based on these guidelines.

### Considerations for future use of the MHFA guidelines for China

#### Repeated suicidal attempts

Evidence suggests that the repetition of suicide attempts is an important societal and health issue among Chinese people, particularly for rural residents and the elderly [7–9, 13]. With a national suicide prevention system yet to be established in China, there are relatively few follow-up health services for people who have attempted suicide [28], though literature show that such services are essential for preventing repeated suicidal attempts [16]. Also, it is not uncommon to believe that suicide is due to weak personality (or not being “tough” enough) and that people should pay for their weakness, without adequate consideration of the multiple risk factors for suicide as well as the need for treatment [1]. Such misconceptions and the scarcity of public services may deter people at risk of suicide from seeking professional help, thus contributing to repeated suicide attempts. Therefore, besides taking an individual’s history of attempted suicide as the warning signs and risk factors for suicide as the English guidelines did, it is needed to include additional actions specifically used to address issues related to repeated suicidal attempts among Chinese people (e.g. to detect long-lasting relationship conflicts, ongoing mental health problems, or inappropriate problem-solving strategy), in order to prevent subsequent attempts and the risk of death from suicide [49].

#### Self-care of suicide first aiders

Despite the fact that the success or failure of a mental health first aid intervention might be influenced by a variety of respondent and recipient factors [50] (e.g. a lack of explicit warning signs, inadequate knowledge and skills to provide help to a person at suicide risk), families and friends frequently feel guilty, painful or confused after a suicide [47]. Furthermore, studies have shown that Chinese families and intimate friends of the suicidal person are likely to ignore their emotional and physical needs and forget that they are also victims of a traumatic event [29, 47]. In comparison with providing mental health first aid for other mental health problems (depression, for example), intervening in a suicidal crisis can be more urgent, complicated and highly emotional, particularly if the person is determined to complete suicide [2]. In the context of a lack of suicide prevention services in China, further consideration of how a mental health first aider might access support is needed, in light of the endorsement of two statements from the guidelines used in English-speaking countries (i.e. *“The first aider must keep in mind that they may not be successful in preventing suicide”* and *“The first aider should exercise appropriate self-care after helping someone who is feeling suicidal”)*.

### Study strengths and limitations

A key strength of this study is that it used a systematic process to ensure the adapted guidelines contain recommendations for providing mental health first aid to a person at risk of suicide that take account of the cultural context and health systems of China. Another strength of the study is the involvement of a diversity of panellists in order to inform better quality expert judgements [31]. In particular, the participation of Hotline operators helped to enrich the diversity of expertise on the panel given their expertise in telephone crisis intervention, which has become an increasingly popular and recognised intervention for a suicidal crisis in China. The relatively large panel sizes (56 and 41 panellists for Round 1 and 2, respectively) also helped to achieve results that are likely to be stable and reliable.

In contrast, only a small number (*n* = 5, out of the 56 experts) of people with lived experience of suicidality or taking care of a suicidal person were involved in this study. This difficulty in the recruitment of people with lived experience of suicidality was also reported in similar studies conducted in other Asian cultures, including India and Sri Lanka [51, 52], which might be related to concerns about stigma and confidentiality and the lack of access to consumer and carer advocacy organisations in these countries. Targeted measures, such as anti-stigma interventions, anonymous participation, and development of relevant advocacy organisations, may contribute to addressing this issue in future studies.

Another limitation of the research findings concerns their generalizability. As panellists in this study were predominantly from urban areas of China with relatively high levels economic development (i.e. Shanghai, Beijing and Jiangsu), the generalizability of the research findings to rural areas and less developed regions in the country is unknown. As these areas have even higher suicide rates, further research should investigate the specific requirements of providing mental health first aid to suicidal persons in these areas. Moreover, the panellists had relatively high levels of education (96% with at least university education) which may also limit the generalizability of the findings to first aiders with less education.

## Conclusions

Through the use of the Delphi method involving local experts in China, we adapted the mental health first aid guidelines for assisting a person at risk of suicide used in English-speaking countries. While very similar to the original guidelines from English-speaking countries, the adapted guidelines also incorporated actions specifically for China including consideration of the person’s attitudes towards suicide, the role of families or friends and actions to remove the means of suicide. The adapted guidelines can be used as a stand-alone product by lay people needing guidance on helping a person at risk of suicide and will be disseminated to suicide prevention and mental health organisations in China. They will also be used to develop the manual and curriculum content of MHFA training in China.

This study potentially creates opportunities for the public to learn basic first aid actions to help a person at risk of suicide, and to implement them when needed, which is an important step towards more effective suicide prevention in China. Further research is needed to investigate the use of these guidelines by the Chinese public. Implementation studies are also warranted in order to explore how the intervention can be tailored to the Chinese context, including its mental health care system, existing workforce and cultural values. The guidelines will also likely need regular updating to take into account of the rapidly changing cultural and health system contexts in China.

## Supplementary information

**Additional file 1.** Statements that were presented to the panel and their ratings across 2 rounds of the survey.

**Additional file 2.** The mental health first aid guidelines for suicide in China.

## Data Availability

The data supporting our findings is attached as the Additional file, which contains all the statements that were presented to panellists and their endorsement rates.

## Abbreviations

LMICs: Low- and middle-income countries
HICs: High-income countries
MICs: Middle-income countries
MHFA: Mental Health First Aid
