# Supplementary material for: Cultural adaptation of the mental health first aid guidelines for assisting a person at risk of suicide to China: a Delphi expert consensus study
Source: BMC Psychiatry. 2020 Sep 16;20:454. doi: 10.1186/s12888-020-02858-9 (PMC7493309; doi:10.1186/s12888-020-02858-9)
Supplement: Supplementary file 2 — Additional file 2. The mental health first aid guidelines for suicide in China. [file 12888_2020_2858_MOESM2_ESM.docx]

**精神健康急救指南 – 自杀篇**

# 指南的目的

指南旨在指导公众如何为可能正在经历自杀问题（包括有自杀想法和自杀行为）的人（在本指南中称为“救助对象”或“对方”）提供初步帮助，即实施“精神健康急救”。

| 精神健康急救(Mental Health First Aid, MHFA): 为发生精神健康问题，或现有精神健康问题恶化，或正在经历精神健康危机(如自杀或创伤经历)的人提供的**初步帮助**，直至对方获得适当的专业帮助或危机解除。 |
| --- |

所谓精神健康急救人员（后简称“急救人员”），即为经历自杀问题的人提供救助的非精神卫生专业人士，他们可以是救助对象的家人、朋友，也可以是同事或邻居，等等。急救人员的作用是为受自杀问题困扰的人提供初步帮助，直至对方获得适当的专业帮助或危机解除。

# 指南的制定

指南的内容是在综合了经验丰富的自杀干预领域专家、曾亲历自杀者和有照顾自杀者经验人士意见的基础上制定的。

# 指南的使用

指南所提供的指导方针仅为一般性建议。每个救助对象的情况不同，急救人员需要根据对方的具体情况对所提供的帮助作出适当调整。

# 关于自杀你应该了解的事实

你需要了解导致自杀的常见危险因素、诱因以及自杀与精神疾病的关系。

| 下列为可能增加自杀风险的部分因素：  - 近期经历较大挫折，一般与人际关系或自身健康有关 - 近期身边有人自杀身亡 - 曾试图自杀或自伤 - 患有身体疾病或残疾 - 患有精神疾病 - 童年时期曾经历身体虐待或性侵犯 |
| --- |

自杀是可以预防的。任何人都可能会产生自杀的想法，但大部分有自杀想法的人并不是真的想要结束生命，他们只是不想伴着痛苦生活。

除非有人告诉你，否则知道某人是否存在自杀想法的唯一方法就是询问。开放地谈论有关自杀的想法和感受，可以挽救生命。

# 如何接近有自杀风险的人

## 接近对方前

不同文化背景下，人们对自杀有不同的看法与态度。自杀精神健康急救人员首先要清楚自己对自杀的看法（例如，认为自杀是错误的，或是理性的选择），以及这些看法对自己提供帮助会产生怎样的影响。

自杀精神健康急救人员需要能够辨识自杀的预警信号。觉察到预警信号后，不要假定对方不需要帮助就会自己好起来，也不要假定他会主动寻求帮助。相反，一旦发现有人想自杀，就要迅速做出反应，比如，向对方的家属或朋友了解他们是否也担心对方存在自杀风险。如果对方已有自伤行为，那么应该首先进行急救处理，并呼叫救护车（电话120）。

| 自杀的常见预警信号：  - 威胁要伤害自己或自杀 - 寻找自杀的方法，如尝试获取药物、刀具或其它工具 - 谈论或写下关于死亡或自杀的内容（如写遗嘱） - 疏远朋友、家人和社会 - 焦虑不安、失眠或嗜睡 - 性情大变 - 表现出绝望 - 感觉失去活着的意义和希望 - 感觉陷入困境、没有出路 - 滥用酒精或违禁药物 - 狂躁、愤怒、寻求报复 - 不计后果、肆无忌惮地行事或从事危险活动   注：想自杀者可能会表现出一个或多个预警信号，也可能表现出列表中未包含的征兆。 |
| --- |

## 接近对方并询问其自杀想法

如果你认为对方可能存在自杀想法，哪怕只是怀疑，也应该直接询问并给予他时间来讨论其消极情绪。如果对方并没有自杀想法，询问本身也不会促使对方产生这个想法，讨论自杀并不会把“自杀”这个想法植入对方脑中。即便他已有自杀想法，询问本身也不会增加其自杀的风险，反而使他有机会谈论自己的问题，并让他感知有人在关心他。

你可以考虑问他现在感觉如何，是否把自己的感受和想法告诉过其他人。你也可以通过描述对方的相关行为来表达自己对他是否会自杀的担心。如果对方急于争辩或非常恼怒，你应该首先安抚他的情绪而非直接提及自杀这一敏感话题，因为这可能会导致对方产生抵触情绪而疏远你。

## 如果感觉对方不愿意与你交谈

当你发现自己无法与对方建立起情感联系，感觉对方可能不想与你交谈时，应该体谅对方，并提出可以找其他人来帮助他。

# 如何评估自杀风险

## 评估紧迫性

你必须认真对待救助对象的自杀想法，并根据所识别的自杀预警信号决定采取行动的紧迫性。即使对方没有自杀计划也不代表他就是安全的。更不要将对方的自杀想法当作是“为了寻求关注”而置之不理。

## 了解对方是否已有自杀计划

近期会将自杀想法付诸实施风险最高的人，是那些有明确的自杀意图、具体的自杀计划的人。因此，你应当询问想自杀者是否有实施自杀的具体计划，包括何时、何地、用何种方法，并且要查明他是否已经采取相应措施来确保其自杀计划的顺利实施。

## 询问其它风险因素

使用酒精或违禁药物会增加一个人把自杀想法付诸行动的风险，因此，你要了解有自杀意图者是否曾经或正在服用某种违禁药物或酗酒。同时，你也应该询问对方是否因为心理健康问题正在接受治疗，或正在服用药物；询问对方近期在工作、社交或家庭生活方面是否发生变故；询问对方是否有过自杀计划或曾自杀未遂，以及他对死亡的态度；询问对方是否有认识的人因自杀而死亡。

# 如何与意图自杀者交谈

## 对自杀想法做出回应

当听到对方告诉你他的自杀想法时，你若感到恐慌或震惊都是正常的，但是，你应该避免把这些情绪（如惊慌、震惊、愤怒等）表现出来。为了能使对方感到安心，你应该表现得镇定自若，并有同理心地对其自杀想法做出反应。允许对方谈论自己的感受，因为说出来以后他可能会感到放松。与此同时，你也要适时提醒对方，不要把自杀想法付诸行动。

## 积极倾听

当有自杀意图者在谈论他的感受时，你应该把全部的注意力都放在他身上，耐心地、不带评判性地听他述说。同时，你也应适时回应并总结他说了什么，以表明自己在听，并向对方澄清重点以确保自己准确地理解。

## 交谈内容

自杀想法往往是一种绝望的挣扎，有自杀意图者试图以此求助、逃避问题或摆脱痛苦，因此，你要允许他发泄，让情绪得以宣泄，例如哭泣、尖叫或愤怒。要询问并允许他谈论自己的想法和感受，比如，想要结束生命的原因、希望通过自杀达到什么具体目的。你应该接纳他的想法和感受，并认可他的这些想法和感受可能很难说出口。但你要让他明白，其实与人谈论这些问题是完全可以的，虽然他自己可能会感到很痛苦。

尽量提出开放性的问题，鼓励想自杀者多说，以便了解更多有关他的想法、感受以及导致其产生自杀想法的原因。在交谈过程中，不要回避使用“自杀”或“自杀死亡”这些词语，救助者不带恐惧和负面评判地直接讨论这个话题很重要。

如果对方说自己听到声音（即出现幻听），那么你应该询问这个声音告诉他什么，或许这个声音与其当前的自杀想法有关。切记，要注意那些可能影响想自杀者当下人身安全的事情。要把重点放在能够保证其安全的事情上，尽量避免那些会使其陷于危险中的事。

## 交谈时应避免什么

- 不要想当然地认为对方的问题并不严重。
- 要时刻注意自己的体态语言，确保不会表现出负面态度或缺乏兴趣。
- 接纳对方说的话，而不是赞成或反对其行为或观点，也不要与对方争辩其自杀想法或讨论自杀的对与错。
- 不要以主导或评判的口吻与对方交谈，如“你不会想做傻事吧”。
- 不要给予肤浅的安慰，如“不要担心”、“振作起来”、“你什么都有”或“一切都会好起来的”。
- 不要使用激将法去刺激对方，如告诉对方“有胆就去做啊”或“你去做呀”。
- 不要使用愧疚感或威胁来阻止对方自杀，如不要说“这样做你会下地狱的”或“你这样会毁了别人的生活”。
- 不要使用带有歧视意味的语言描述自杀，如将自杀称为“寻短见”或将自杀未遂称为“失败”或“不成功”。
- 不要因害怕说错话或担心说得不恰当而不鼓励对方说话，你要明白，询问对方的自杀想法比斟酌用词更重要。
- 不要打断对方来谈自己的感受或经历。
- 不要试图为对方诊断其心理问题。
- 不要受对方情绪影响，把对方任何伤感情的行为或言辞当作是针对自己的。
- 不要尝试为对方承担责任。

## 让对方知道你关心他并使其感到安心

你要让意图自杀者知道你对他的关心，并表现出理解和支持，表示愿意帮助他，例如，询问对方想要得到怎样的帮助、自己可以具体为他做些什么。让对方放心，不论他说什么你都愿意听。你可以告诉他，自杀想法其实很普遍，很多人在一生中的某个阶段都会有这种想法。告诉他除了自杀，还有其它解决问题的方法和途径。

切记，由衷地关心并真诚地想要帮助对方比说大道理或讲述自己与对方有相似的背景更重要**。**

## 强调积极因素

你应当向对方指出，他之所以还活着并且能够跟你谈论他的感受，说明他可能对是否要实施自杀并不太确定。多与对方讨论其生命中“美好的事情”、对未来的希望以及活下去的其它理由。尝试找出过去是什么力量在支撑着他以及这些力量是否依然存在。要感谢对方能与自己分享他的感受和想法，并认可这是需要勇气的。

# 如何提供初步帮助

如果发现救助对象有自杀想法，尽量不要留其独处，但在安全的环境下，可以考虑给予对方适当的独处时间。

尝试找出对意图自杀者有用的资源和服务信息并告知对方，比如，通过询问他现在的居住情况（独居、和父母一起住、和朋友一起住等）来判断他身边是否有可用的社会支持。也可以直接询问对方现有的可以帮到他的资源（如，家人、朋友或其他可以信赖的家属）。

不要独自行动来防止其自杀，尽量通过与意图自杀者合作来保证其安全，例如，可以通过讨论的方式来决定采取什么行动以获得帮助。

尽快获得适当的专业帮助（即去看精神科医生或找心理治疗师）很重要，即使对方不愿意甚至可能会因为你试图阻止他自杀或帮助其获得专业协助而表现出愤怒或感到遭受背叛，你仍然要坚持鼓励其接受专业治疗。同时，你也要了解对方不愿寻求帮助的原因。如果需要联系医务人员，最好联系对方认识和信任的医生。如果对方不愿与人面对面交流，则应鼓励其拨打当地的心理援助热线。

如果怀疑对方有把自杀想法立即付诸行动的风险（比如已有具体计划），即使不确定也要迅速采取行动，比如，致电当地精神卫生中心或心理援助热线咨询对于当前情况的处理办法。如果对方不愿做出保证安全的承诺，你应在获得对方允许后，联系其平时看的精神科医生或心理治疗师，并询问他们的意见。如果对方已有实施自杀计划的具体方法，如，已准备好自杀工具，你应在征得对方同意后拿走这些工具。即使对方不同意，还是应在可能的情况下，把工具拿走。

在救助过程中遇到困难时（如，对方拒绝专业帮助、拒绝交出自杀工具），可以致电当地精神卫生中心或心理援助热线，咨询其对当前情况的建议。在对方持有武器的情况下，你应该考虑报警，并告知警察当事人有自杀倾向，以提醒他们适当应对。

在为意图自杀者提供帮助时你要注意：

- 不要将自己置于危险之中。
- 虽然为对方提供帮助，但你对对方的行为或行动不负有责任，并且也不能控制他人决定要做的事。
- 不要低估自己帮助意图自杀者的能力；同时，也不要高估自己，因为你可能最终无法阻止对方自杀。
- 在帮助意图自杀者后，你应该考虑进行适当的自我调节。

# 制订安全计划

## 内容

安全计划应该清晰明确，讲清楚将要完成什么、由谁来做以及何时做。内容应更多地集中在对方应该做什么，而不是不该做什么。安全计划的制订，其持续的时间对对方来说应当是容易做到的，以便使其能够遵守约定，从而获得成就感。尽量让对方参与共同制订安全计划，并且可以要求他对于不自杀计划做出书面或口头的承诺。你应该将安全计划的内容整理成清单，放在意图自杀者容易拿到的地方。

注意，不要以为有了安全计划就能够保障意图自杀者的安全。

## 安全联系人

安全计划应该包括24小时安全联系人的联系方式，包括家人、朋友、意图自杀者的医生和心理援助热线，并且要让其把安全联系人的名单随时带在身边，如果感觉自己想自杀，就立刻拨打电话给安全联系人。

# 能否答应对自杀风险保密

绝不应同意为自杀风险以及自杀者的自杀计划保密，更不应为对方的严重犯罪行为或意图保密。但是，要尊重意图自杀者，与其共同决定还应告诉谁他可能会自杀。如果对方不同意向其他人透露其自杀想法，为了确保他的安全，你可能需要违背他的意愿。在这种情况下，你应先向对方解释为什么，比如，“你需要帮助，我是来帮你求助的，因此不能保守这样的秘密” ，然后坦率地告诉对方将要通知谁。但注意不要将告知范围扩大化，仅告知必要的相关人员即可（如，意图自杀者的合法监护人、可提供帮助的专业机构、警方等）。切记，宁可在未经当事人许可的情况下透露其自杀想法以获得帮助，让对方怨恨自己，也不能任凭其自杀行为得以实施而失去生命。
